# Association between statin use and osteoporotic fracture in patients with chronic obstructive pulmonary disease: a population-based, matched case-control study

**DOI:** 10.1186/s12944-020-01412-6

**Published:** 2020-11-03

**Authors:** Hung-Yi Chen, Pei-Yu Su, Tsung-Kun Lin, Gwo-Ping Jong

**Affiliations:** 1 Department of Pharmacy, China Medical University, Taichung, Taiwan, Republic of China; 2 Department of Pharmacy, China Medical University Beigang Hospital, Yunlin County, Taiwan, Republic of China; 3 Department of Pharmacy, Wan Fang Hospital, Taipei Medical University, Taipei, Taiwan, Republic of China; 4 Department of Pharmacy, Taoyuan Armed Forces General Hospital, Taoyuan, Taiwan, Republic of China; 5 School of Pharmacy, National Defense Medical Center, Taipei, Taiwan, Republic of China; 6 Department of Internal Medicine, Chung Shan Medical University Hospital and Chung Shan Medical University, Taichung, Taiwan, Republic of China

**Keywords:** Chronic obstructive pulmonary disease, Osteoporosis fracture, Statin, Dyslipidemia, Case-control study, Cohort study, Taiwan

## Abstract

**Background:**

In the recent years, chronic obstructive pulmonary disease (COPD) has been found to be associated with a higher risk of new-onset osteoporotic fracture (NOF). However, the existence of such an association in the COPD patients receiving statin treatment remains unknown. The present study aimed to investigate the association between COPD and NOF in statin-treated patients.

**Methods:**

The present study was conducted over a period of 10 years (January 2004 to December 2013) in Taiwan. COPD patients receiving statin treatment were included in the statin user group, whereas the randomly selected statin non-users, with 1:1 matching for sex, age, index date, and Charlson Comorbidity Index, were included in the statin non-user group. The hazard ratio (HR) of NOFs in COPD patients was estimated between statin user and non-user groups.

**Results:**

A total of 86,188 cases were identified as the statin-treated patients, and 86,188 subjects were included in the control group of statin non-users. Initially, the risk of NOF was found to be higher among the statin users as compared to non-users [HR, 1.12; 95% confidence interval (CI), 1.01–1.25]. However, the calculation of risk for NOFs after the adjustment for age, sex, comorbidities, and concurrent medications indicated no association of NOF (HR, 0.81; 95% CI, 0.55–1.21) with COPD in patients receiving statin treatment as compared to statin non-users.

**Conclusion:**

The results of the study provided first evidence for the absence of any association between COPD and NOFs in statin-treated patients during a follow-up period of 10 years. Thus, the findings of this study might support the hypothesis stating the potent pleiotropic effects of statins. In clinical practice, these drugs might prove beneficial for the patients with COPD.

## Background

In the past few decades, chronic obstructive pulmonary disease (COPD) has emerged as the most common chronic respiratory disease worldwide [1]. It is a chronic lung disease, characterized by difficulty in breathing and obstructed air flow. According to the global estimates of 2016, COPD affected approximately 251 million adults and ranked third among the leading causes of death in adults [2]. The global prevalence and mortality related to COPD is expected to increase in coming years owing to an increase in the number of cigarette smokers. In fact, COPD is projected to emerge as the third leading cause of death by 2020 [3, 4]. Globally, there has been a gradual increase in the concerns related to healthcare for COPD, especially for comorbidities related to it.

In 2010, the global prevalence of osteoporotic fracture in patients aged 50 years or more was recorded to be ~ 158 million. These incidences of osteoporotic fracture are expected to double by 2040 [5]. Osteoporotic fractures are associated with significant healthcare costs as they have been shown to adversely affect the overall quality of life [5]. Thus, patients with COPD and osteoporotic fractures account for significant healthcare expenditures for the sexes, making them a major global public health challenge [6, 7].

Several previous studies have reported an association between the use of steroids and risk of new-onset osteoporotic fractures (NOFs) in patients with COPD [8, 9]. In particular, an increased risk of NOF was observed, especially in case of chronic steroid user, suggesting a direct osteoporotic effect of steroids [10, 11]. This association of steroids with osteoporosis and osteoporotic fractures might be contributed by promotion of apoptosis of both osteoblasts and osteoclasts and decrease in their recruitment from progenitor cells [11]. In comparison to this, statins might influence bone metabolism by increasing the formation of bones and decreasing the risk of NOF [12]. Recently, several prospective and retrospective studies have investigated the association between COPD and NOF in statin-treated patients [13–15]. However, the outcome of these studies was limited by small sample size and insufficient follow-up periods [16, 17]. Notably, the association between COPD or its prescribed drugs and a higher risk of NOF in statin-treated patients with dyslipidemia over a long-term follow-up remains unclear. The present study aimed to investigate the association between COPD and NOF in statin-treated patients in Taiwan, over a period of 10 years.

## Methods

### Study designs and cohort populations

A population-based case–control study was conducted and used data obtained from claim forms submitted to the Taiwan National Health Insurance Research Database (NHIRD) between 2004 and 2013. The National Health Insurance program was established in 1995 in Taiwan and currently has a coverage rate of 99%.

The Longitudinal Health Insurance Research Database (LHIRD) 2010 was used in this study and comprised a systematic and random sampling of the NHIRD for one million beneficiaries. Patients were included in the study if they had COPD [International Classification of Diseases, Ninth Revision, Clinical Modification, (ICD-9-CM) code 491)] and used statins without osteoporotic fracture at baseline (January to March, 2004). We summarized the claim records of each patient into one record. Participants were defined as having new-onset osteoporotic fracture (ICD-9-CM codes 733.11 and 805–829) or osteoporosis (ICD-9-CM code 733.11) with fracture-related surgery (ICD-9-CM codes 78.1, 78.4, 78.5, 78.9, 79, and 81) and COPD between January 01, 2004 and December 31, 2013. Patient were excluded as follows: (1) age < 40 years at baseline; (2) had a prior history of osteoporotic fracture (ICD-9-CM codes 733.11 and 805–829) or osteoporosis (ICD-9-CM code 733.11) with fracture-related surgery (ICD-9-CM codes 78.1, 78.4, 78.5, 78.9, 79, and 81) before January 01, 2004; (3) were taking calcium supplements, bisphosphonates, raloxifene, teriparatide, or calcitonin between 2004 and 2013; (4) had pathological fractures (ICD-9-CM codes 805–829) resulting from cancer metastasis (ICD-9-CM codes 140–239), renal osteodystrophy (ICD-9-CM codes 588.0), or secondary hyperparathyroidism (ICD-9-CM codes 588.81) at baseline; and (5) were experiencing other major medical problems that would leave the patient with a life expectancy of < 6 months (die between January 2004 and July 2004). The NOF was defined as the first time that an osteoporotic fracture or osteoporosis with fracture-related surgery code appeared in the claim record. The main outcome was NOF diagnosis after the baseline date. Both cases and controls were matched 1: 1 for sex, age (5-year intervals), index date, and Charlson Comorbidity Index score.

### Statistical analyses

Data are presented as valid percentages and mean values with standard deviations. Chi-square tests and *t*-tests were used for univariate analyses. Cox proportional hazards regression models were applied to calculate hazard ratio (HR) and 95% confidence interval (CI) for the association between osteoporotic fracture and statin use in patients with COPD. Additional adjusted multiple Cox proportional hazards regression models, including sex, age, comorbidity, and concurrent medications, were implemented. Finally, the NOF-free survival rates between COPD patients in the statin-treated group and without statin-treated group were estimated by the Kaplan–Meier method using the log rank test. A *P* < 0.05 was considered statistically significant. All statistical calculations were performed with Statistical Analysis software, version 9.3 (SAS Institute, Inc., Cary, NC, USA).

## Results

### Patients’ baseline characteristics

**Fig. 1:**
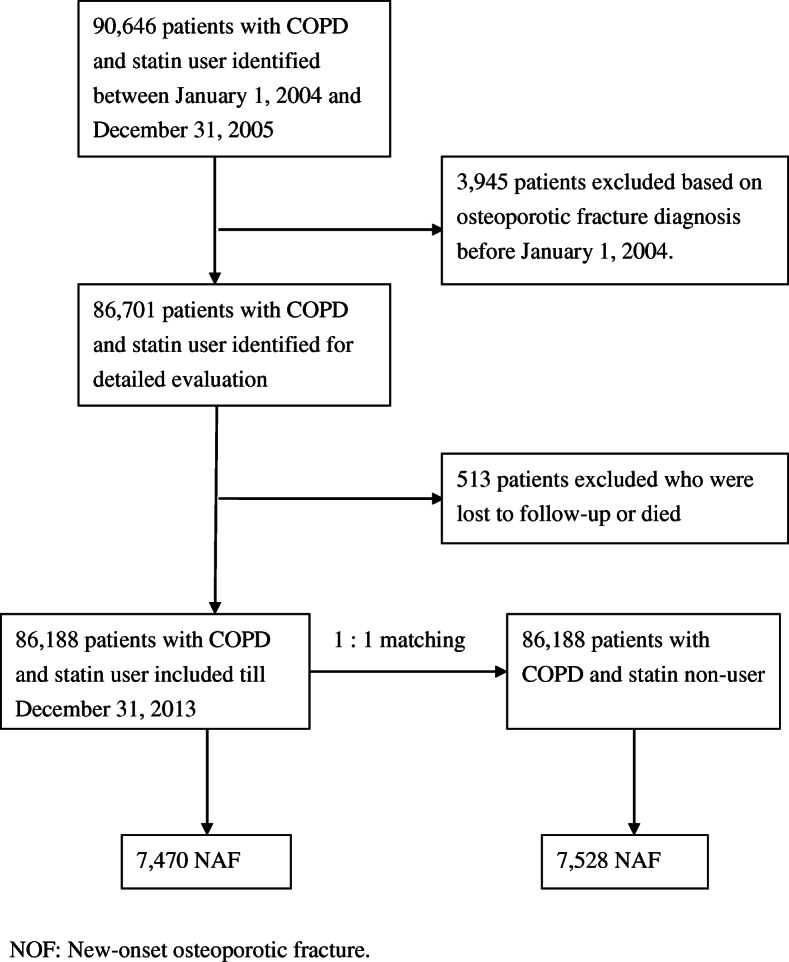
Flowchart of selection of patients for the inclusion in this study

Between 2004 and 2005, 90,646 patients with COPD were identified from the National Health Insurance Research Database (*n* = 1,000,000). Among these, 3945 patients were excluded based on the diagnosis of osteoporotic fracture before January 01, 2004; 480 patients were lost to follow-up, and 33 patients died. A total of 86,188 patients with COPD and statin user were selected for this study. Another 86,188 patients with COPD and statin non-user, who were assigned to non-user group after 1: 1 sex-matched, age-matched, index date-matched, and Charlson Comorbidity Index score-matched randomly selected participants (Fig. 1). Baseline characteristics, comorbidities, and concurrent medication use between the statin user group and statin non-user groups in patients with COPD are presented in Table 1. The mean ages of patients with and without statin-treated were 64.4 and 64.5 years, respectively. Study participants were predominantly women (54.4%).

**Table 1:** Baseline characteristics of all patients

	Statin user (*N* = 86,188)	Statin non-user (*N* = 86,188)	*P* value
Age (year-old)	64.4 ± 12.9	64.5 ± 13.1	0.998
Sex			1.000
Male (%)	39,302 (45.6)	39,302 (45.6)	
Female (%)	46,886 (54.4)	46,886 (54.4)	
Index date (years)	4.5	4.5	1.000
Charlson score (Mean + SD)	4.1 + 1.0	3.8 + 0.9	0.902
Comorbidities (n, %)			
Hypertension (%)	19,909 (23.1)	10,170 (11.8)	< 0.001
CAD (%)	3706 (4.3)	1724 (2.0)	< 0.001
CHF (%)	371 (0.4)	259 (0.3)	0.663
CKD (%)	3017 (3.5)	1034 (1.2)	< 0.001
DM (%)	6206 (7.2)	2758 (3.2)	< 0.001
Dementia (%)	172 (0.2)	431 (0.5)	0.526
Cancer (%)	1724 (2.0)	2591 (3.0)	0.450
Hyperthyroidism (%)	172 (0.2)	1120 (1.3)	0.011
Obesity (%)	259 (0.3)	2155 (2.5)	0.005
Depression (%)	1034 (1.2)	948 (1.1)	0.978
Rheumatoid arthritis	690 (0.8)	690 (0.8)	1.000
Alcoholism (%)	171 (0.2)	431 (0.5)	0.669
Concurrent medications (n)			
Loop diuretics (%)	9912 (11.5)	5171 (6.0)	< 0.001
Thiazide diuretics (%)	4482 (5.2)	2155 (2.5)	< 0.001
Beta-blockers (%)	5731 (6.5)	5688 (6.6)	0.870
DHP CCBs (%)	10,429 (12.1)	9308 (10.8)	0.661
Non-DHP CCBs (%)	3275 (3.8)	1551 (1.8)	< 0.001
Alph-blockers (%)	1551 (1.8)	517 (0.6)	< 0.001
ACE inhibitors (%)	3189 (3.7)	3792 (4.4)	0.047
ARBs (%)	8274 (9.6)	8188 (9.5)	0.916
Benzodiazepines (%)	25,253 (29.3)	24,908 (28.9)	0.330
SSRI (%)	862 (1.0)	30 (0.0)	< 0.001
Glucocorticoids (%)	29,304 (34.0)	24,564 (28.5)	< 0.001
Opioid analgesics (%)	5430 (6.3)	5688 (6.6)	0.222
HRT (%)	603 (0.7)	1207 (1.4)	< 0.001

*NOF* New-onset osteoporotic fracture, *CAD* Coronary artery disease, *CHF* Congestive heart failure, *CKD* Chronic kidney disease, *DM* Diabetes millitus, *DHP CCBs* Dihydropyridine calcium channel blockers, *ACE* Angiotensin converting enzyme, *ARBs* Angiotensin receptor blockers, *SSRI* Selective serotonin reuptake inhibitors, *HRT* Hormone replacement therapy, *SD* Standard deviation

*p* value between NOF in statin-treated group and non-NOF without statin-treated group

Hypertension and diabetes mellitus were the most commonly observed comorbidities. Except for congestive heart failure, dementia, cancer, depression, and alcoholism, all other comorbidities, such as hypertension, coronary artery disease, chronic kidney disease, diabetes mellitus, hyperthyroidism, rheumatoid arthritis, and obesity, were significantly different between the statin user group and the statin non-user group (*P* < 0.05). The distribution of prescriptions, including loop or thiazide diuretics, nondihydropyridine calcium channel blockers, alpha-blockers, ACE inhibitors, selective serotonin reuptake inhibitors, glucocorticoids, and hormone replacement therapy, was also significantly different between NOF group and the non-NOF group (*P* < 0.05; Table 1).

### Relative risk of NOF in COPD patients with statin use

**Fig. 2:**
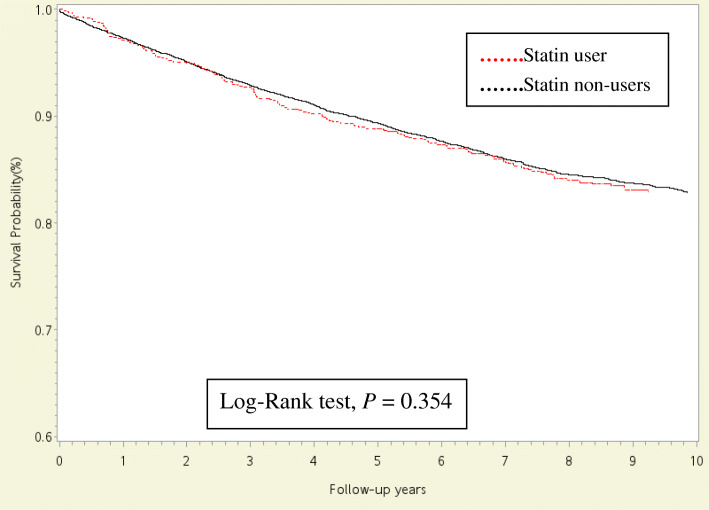
Kaplan-Meier analysis comparing probabilities of free from new-onset osteoporotic fracture between COPD patients with statin-treated and without statin-treated

Initially, the crude OR of NOFs was higher among patients with COPD in the statin-treated group (HR, 1.12; 95% CI, 1.01–1.25) than that among patients with COPD in the without statin-treated group. However, the risk for NOFs was not associated with COPD after adjusting for age, sex, other comorbidities, and concurrent medications (adjusted OR, 0.81; 95% CI, 0.55–1.21; Table 2). Finally, the Kaplan–Meier survival analysis revealed no difference in the probabilities of free from NOFs between patients with COPD with statin user and those with COPD in the without statin-treated group (*P* = 0.354; Fig. 2).

**Table 2:** Incidence of hazard ratios (HRs) with 95% confidence intervals (CIs) for new-onset osteoporotic fracture in statin-treated patient COPD compared with statin non-user COPD subjects

	Crude β (95% CI)	Crude HR (95% CI)	*P* value	Adjusted β^a^ (95% CI)	Adjusted HR^a^(95% CI)	*P* value
COPD without statin-treated	ref	ref		ref	ref	
COPD in statin-treated	0.113 (0.010 to 0.223)	1.12 (1.01–1.25)	0.032	−0.211 (−0.598 to 0.191)	0.81 (0.55–1.21)	0.584

^a^(β and HR) were adjusted for age, sex, hypertension, CAD, CHF, CKD, DM, dementia, cancer, hyperthyroidism, obesity, depression, rheumatic arthritis, alcoholism and concurrent medication

### Discussion

The results of the present study revealed that statin use was not independently associated with an increased risk of NOF in patients with COPD. Statin use might provide some beneficial effects on NOF in statin-treated patients with COPD.

A significantly higher prevalence of osteoporotic fractures has been previously reported in patients with COPD as compared to healthy subjects, particularly in females [18–20]. The presence of an association between COPD and NOF has been previously reported in many studies. A cross-sectional study conducted by Watanabe et al. reported a higher prevalence (79.4%) of osteoporotic vertebral fracture in Japanese men with COPD [18]. In a similar study, Reyes et al. reported an independent association between COPD and an increased risk of hip NOF in Catalonians [19]. However, none of the aforementioned studies involved a prospective study design. Dam and co-workers conducted a prospective study to investigate the association between COPD or asthma and NOF [14]. The results of the study revealed that male patients with COPD or asthma were associated with 42 and 164% higher risk of sustaining non-vertebral and vertebral NOF, respectively. In contrast to this, the results of the present study demonstrated absence of a significant association between risk of NOF and COPD in statin-treated patients. These differences in the results of the present study as compared to the previous studies might be attributed to differences in the baseline characteristics of the patients (one study included general population, whereas three studies included only men), study design (one study involved a prospective design, while three studies included a retrospective design), and statin use [14, 18–20].

In the present study, the association between COPD and NOF in statin-treated patients could be attributed to the shared risks associated with other conditions, such as smoking, physical inactivity, uncontrolled inflammation, and dyslipidemia [14, 20]. An independent association between statin use and lower risk of NOF has been previously demonstrated [21–23]. Statins are one of the most effective agents used in the control of dyslipidemia and prevention of cardiovascular diseases [24, 25]. In addition to this, statins might influence bone metabolism by increasing bone formation [12]. In the present study, all the patients with dyslipidemia and COPD received statin therapy, which might have incurred a beneficial effect with regard to the risk of NOF.

In the present cohort study, COPD patients with dyslipidemia and other comorbidities, such as chronic kidney diseases, diabetes mellitus, and dementia, showed a higher risk of developing NOF as compared to the patients devoid of these comorbidities. Several previous studies, both experimental and those involving humans, have demonstrated a deteriorative effect of chronic kidney disease, diabetes mellitus, and dementia on bone metabolism [26–30]. In a similar study conducted by Reyes et al., the conditions of chronic kidney disease, diabetes mellitus, or dementia were found to be independently associated with a higher risk of hip fracture in older men [19].

The association between COPD and higher risk of NOF has been reported in several previous studies [8, 9]. An increased risk of NOF has been observed in COPD subjects, especially in elderly patients receiving steroid therapy, suggestive of a potential direct osteoporotic effect [10, 11]. In the present study, all the patients with dyslipidemia and COPD received statin therapy, which might have incurred a beneficial effect on the risk of NOF. Therefore, the present study proposes to include statins as an integral part of the prevention strategy for osteoporotic fractures in patients with COPD in clinical practice, and it should not be restricted only to the treatment of COPD and dyslipidemia.

### Study strengths and limitations

This study has two strengths. First, this is the first evidence regarding the neutral association between statin use and NOFs in patients with COPD during a 10-year follow-up. Second, this is a population-based cohort study. Some limitations of this study need to be emphasized. First, all cases in this study were collected from claim forms of the NHIRD, and the diagnoses were based on physician reports only. Therefore, it is unclear how our findings can be generalized to patients in different regions of the world. Second, this was a descriptive, retrospective study conducted in Taiwan for over 10 years. Moreover, we excluded irregularly treated COPD patients from the analyses. Therefore, caution must be exercised in interpreting the results of this study. Third, the risk factors for osteoporotic fractures, such as obesity, body mass index, smoking status, alcohol consumption, physical activity, family history, as well as the degree and duration of the disease were not available from these secondary data. Fourth, we determined that the exposure to statins in the cohort is real, given that the claims data include medication prescriptions. However, laboratory data, treatment adherence, lifestyle adjustments and modifications, history of treated liver disease, history of resolved vitamin D deficiency, improved quality of life, increased physical activity, reduced stress, treated gastrointestinal tract disorders, and other health-related factors were not available from these secondary data. However, because the data we used were population-based data, we assumed that there were no differences among the two groups.

### Conclusions

In conclusion, the results of the present study indicated that statin therapy in COPD patients was not independently associated with an increased risk of NOF. Further, these findings might support the hypothesis stating the potent pleiotropic effects of statins. In fact, the use of these drugs might prove beneficial for patients with COPD in clinical practice.

## Data Availability

The raw data supporting the conclusions of this manuscript will be made available by the authors. The data of this study including figures and tables will be available by contacting corresponding author. The data can be used permanently after the article is published.

## Abbreviations

ACE: Angiotensin-converting enzyme
CI: Confidence interval
COPD: Chronic obstructive pulmonary disease
ICD-9-CM: International Classification of Diseases, Ninth Revision, Clinical Modification
HR: hazards ratio
LHIRD: Longitudinal Health Insurance Research Database
NHIRD: National Health Insurance Research Database
NOF: New-onset osteoporotic fracture.
